# Development of a Convolutional Neural Network-Based Colonoscopy Image Assessment Model for Differentiating Crohn’s Disease and Ulcerative Colitis

**DOI:** 10.3389/fmed.2022.789862

**Published:** 2022-04-08

**Authors:** Lijia Wang, Liping Chen, Xianyuan Wang, Kaiyuan Liu, Ting Li, Yue Yu, Jian Han, Shuai Xing, Jiaxin Xu, Dean Tian, Ursula Seidler, Fang Xiao

**Affiliations:** ^1^Department of Gastroenterology, Tongji Hospital, Tongji Medical College, Huazhong University of Science and Technology, Wuhan, China; ^2^Wuhan United Imaging Healthcare Surgical Technology Co., Ltd., Wuhan, China; ^3^Department of Gastroenterology of Hannover Medical School, Hanover, Germany

**Keywords:** inflammatory bowel disease, Crohn’s disease, ulcerative colitis, artificial intelligence, deep learning, convolutional neural network, colonoscopy image, classification

## Abstract

**Objective:**

Evaluation of the endoscopic features of Crohn’s disease (CD) and ulcerative colitis (UC) is the key diagnostic approach in distinguishing these two diseases. However, making diagnostic differentiation of endoscopic images requires precise interpretation by experienced clinicians, which remains a challenge to date. Therefore, this study aimed to establish a convolutional neural network (CNN)-based model to facilitate the diagnostic classification among CD, UC, and healthy controls based on colonoscopy images.

**Methods:**

A total of 15,330 eligible colonoscopy images from 217 CD patients, 279 UC patients, and 100 healthy subjects recorded in the endoscopic database of Tongji Hospital were retrospectively collected. After selecting the ResNeXt-101 network, it was trained to classify endoscopic images either as CD, UC, or normal. We assessed its performance by comparing the per-image and per-patient parameters of the classification task with that of the six clinicians of different seniority.

**Results:**

In per-image analysis, ResNeXt-101 achieved an overall accuracy of 92.04% for the three-category classification task, which was higher than that of the six clinicians (90.67, 78.33, 86.08, 73.66, 58.30, and 86.21%, respectively). ResNeXt-101 also showed higher differential diagnosis accuracy compared with the best performing clinician (CD 92.39 vs. 91.70%; UC 93.35 vs. 92.39%; normal 98.35 vs. 97.26%). In per-patient analysis, the overall accuracy of the CNN model was 90.91%, compared with 93.94, 78.79, 83.33, 59.09, 56.06, and 90.91% of the clinicians, respectively.

**Conclusion:**

The ResNeXt-101 model, established in our study, performed superior to most clinicians in classifying the colonoscopy images as CD, UC, or healthy subjects, suggesting its potential applications in clinical settings.

## Introduction

Inflammatory bowel disease (IBD) is a chronic and progressive inflammatory condition characterized by a relapsing and remitting course of inflammation of the gastrointestinal tract lining. IBD mainly includes two forms of diseased conditions, namely Crohn’s disease (CD) and ulcerative colitis (UC). Internationally, the prevalence of IBD is increasing at an alarming rate, especially in the developed and industrialized countries (1, 2).

Accurate identification and differential diagnosis of IBD remains a challenge due to the involvement of several disease influential factors, such as increasing prevalence of intestinal infections, absence of a universal diagnostic standard, overlapping clinical manifestations with respect to non-IBD gastrointestinal disorders (3), etc. Often, it becomes very challenging to diagnose between CD and UC differentially, as both of them involve multi-factorial parameters, such as medical history, clinical manifestations, laboratory findings, radiological examinations, histopathology, and endoscopy (4–6). Amongst them, endoscopic evaluation plays a key role in the effective diagnosis, management, prognosis, and surveillance of IBD patients (7). However, in such cases, except for repetitive and arduous manual operations, subjectivity is still one of the major drawbacks that greatly depend on knowledge, professional experience, and perceptual factors of the clinician performing the procedure (8–10). Thus, developing intelligent auxiliary tools is of immense importance for efficiently and quickly processing tons of medical data, not only to practically overcome the above shortcomings but also to gear up the IBD surveillance globally. In fact, several researches have shown artificial intelligence (AI) could partially make up the deficiency caused by clinicians, and it is expected to support lesion recognition and guide therapeutic decision making by providing feedback (11, 12).

Notably, AI with deep learning-guided high-capacity image recognition has successfully been applied and clinically tested in the diagnosis and classification of various diseases, including skin cancer, premature retinopathy, large vessel occlusion of CT angiography detection (13–15), etc. More recently, a convolutional neural network (CNN), an end-to-end deep learning system in combination with pattern recognition, feature extraction, and classification, has opened the door to elaborate image analysis (16–18).

For gastroenterological disorders, AI has been widely used to recognize early esophageal neoplasia in Barrett’s esophagus, classify gastric cancers and ulcers, localize and identify polyps (19–21), etc. The proposed computer-aided endoscopic diagnosis systems have shown potential advantages in reducing manual workload as well as undesired human error and improving the accuracy of medical diagnosis. However, the clinical application of deep learning has rarely been investigated in training a CNN model to analyze, interpret and extract characteristic features of IBD from colonoscopy images to achieve the distinction of CD, UC, and healthy controls. Therefore, the primary aim of the present study was to apply an AI-guided image analysis model for classifying CD, UC, and normal gastrointestinal conditions at the endoscopy image level.

## Materials and Methods

### Study Subjects

We retrospectively searched the in-patient medical record database for subjects undergoing colonoscopy between January 2014 and May 2021 at three campuses of Tongji Hospital, Tongji Medical College, Huazhong University of Science and Technology. The inclusion criteria for enrolling CD/UC patients were as follows: (1) the clinical diagnoses were made *via* a combination of clinical, laboratory, endoscopic, and histological criteria according to the third European Crohn’s and Colitis Organization (ECCO) consensus (5, 6), (2) CD/UC patients in the active stage under the endoscopy. Exclusion criteria were: (1) ileocolectomy was performed before colonoscopy, (2) colonoscopy revealed no active lesion, (3) CD patients with lesions restricted to the ileum. Included healthy controls were adults who showed no abnormalities in the health check-up. Endoscopic examination was performed using CF-H260AI, CF-Q260AI, CF-H260AZI, CF-H290I, or CF-HQ290I endoscope (Olympus Optical Co., Ltd., Tokyo, Japan).

For estimating the sample size, we referred to the content of “a supervised deep learning algorithm will generally achieve acceptable performance with around 5,000 labeled examples per category” recorded in the Deep Learning textbook written by Goodfellow et al. on page 24 (22). Generally speaking, 60–80 images would be captured during one colonoscopy, of which the images of the lesion area of a CD/UC patient account for about 20–40%, and the colonic images of a healthy control account for about 70–80%. To meet the size requirement of approximately 5,000 eligible examples per category, we set the sample sizes of about 200 CD/UC patients and 100 healthy controls based on the data availability.

### Image Collection Procedure

Conventional white light colonoscopy images captured from included CD patients, UC patients, and healthy controls were enrolled for this study. Before being fed to the deep learning model, multiple preprocessing operations can be performed on raw image data to reorganize them into a uniform format: (1) Data exclusion: non-informative images were excluded. (2) Boundary cropping: the black margin surrounding the colonoscopy image with date and time of acquisition was cropped by software. (3) Resizing: images were resized to a standard resolution of 256 × 256 to fit the expected size for model training. (4) Horizontal flipping: images were flipped horizontally for data augmentation. (5) Normalization: gray-scale normalization was performed to increase the rate of convergence of the network. Specifically, non-informative images were excluded according to the following exclusion criteria: (1) images of poor quality, (2) poor bowel preparation, (3) fragmented images of the small intestine, (4) inactive IBD lesion, and (5) CD/UC images with normal endoscopic features (Supplementary Figure 1).

**FIGURE 1:**
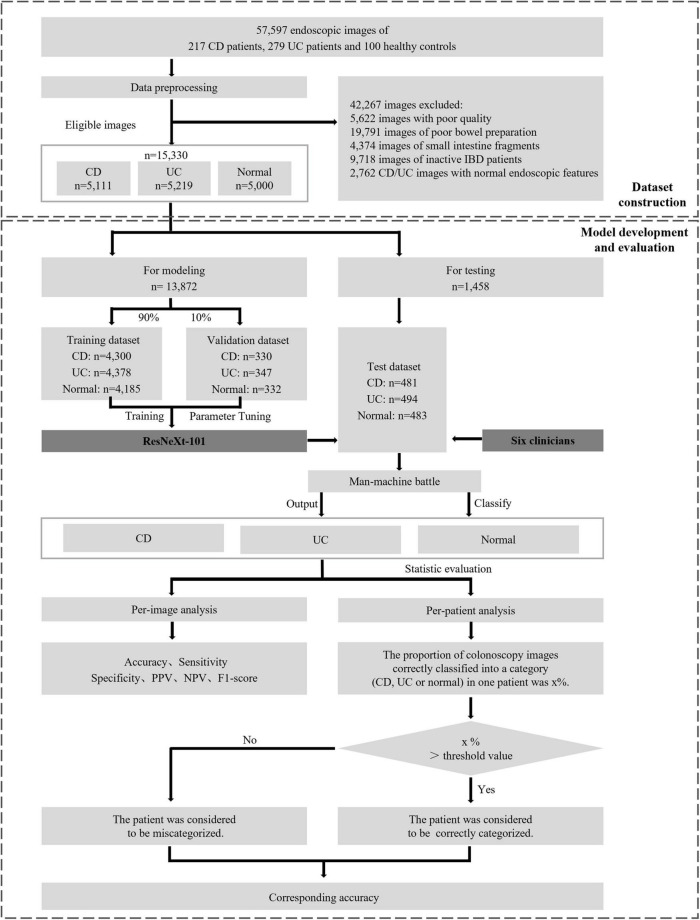
Overall study design. The main processes involved are eligible colonoscopy image set construction, model development, and final evaluation the performance between CNNs and clinicians. CD, Crohn’s disease; UC, ulcerative colitis; IBD, inflammatory bowel disease; PPV, positive predictive value; NPV, negative predictive value.

The randomization was performed in the CD image set, UC image set, and normal image set, respectively. Within these sets, images were divided into the training phase and testing phase, while the former one is further partitioned into a training dataset consisting of approximately 90% of the images and a validation dataset consisting of the remaining 10%. The overall experimental design, dataset selection, and distribution procedures are presented in Figure 1. The study was conducted in accordance with the Declaration of Helsinki and approved by the Ethical Committee of Tongji Hospital.

### Training of the Convolutional Neural Network Model

**FIGURE 2:**
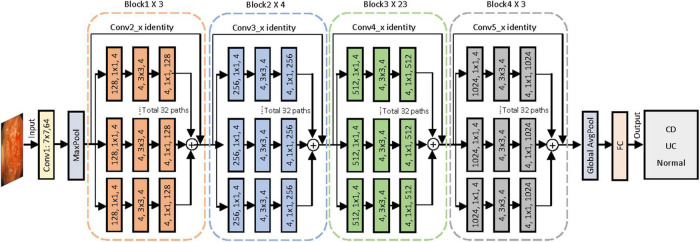
Proposed convolutional neural network for colonoscopy image classification with ResNeXt-101 residual network architecture. A layer is shown as (# in channels, filter size, # out channels). Conv, convolutional layer; AvgPool, average pool; FC, full connected layer; CD, Crohn’s disease; UC, ulcerative colitis.

To construct the AI-based classification system, we selected the CNN architecture of ResNeXt-101 after measuring the performances of five different networks, namely, ResNet-50, ResNet-101, ResNeXt-50, ResNeXt-101, and EfficientNet-V2. ResNeXt-101 was a deep CNN pre-trained with data from ImageNet and then re-trained using our image set by fine-tuning the parameters of all layers. Figure 2 shows the entire architecture of ResNeXt-101. The linear rectifying unit activation function is implemented to all the convolution layers, followed by the batch normalization. The network enters the fully connected layer through the average pooling layer and outputs the classification category through the Softmax function. Through the shortcut connection, which encourages the feature reuse to reduce the feature redundancy, the ResNeXt-101 can address the problem of accuracy of classification tasks that tend to be saturated or even degraded as the network deepens (23). The ResNeXt-101 network reached the highest accuracy after running a total of 300 training epochs with a batch size of 64. Eventually, the model yielded the categorical classification of each of the input endoscopic images as CD, UC, or normal with the maximum probability in the output.

### Outcome Measures and Statistical Analysis

Using a test image set of 1,458 images, the classification performance of the constructed CNN model was evaluated and compared with that of six clinicians of different endoscopic operation experiences. Clinicians were blinded to any relevant information about the test image set and classified these images independently.

The per-image analysis was defined as per-image diagnosis of CD, UC, or normal. Evaluation indicators, including the accuracy, sensitivity, specificity, positive predictive value (PPV), negative predictive value (NPV), and F1-score of the classification capability of CD, UC, or normal images, were compared. F1-score was calculated as follows:


$$\mathrm{F1}-\mathrm{score}=\frac{2*\mathrm{true}\,\mathrm{positive}}{2*\mathrm{true}\,\mathrm{positive}+\mathrm{false}\,\mathrm{positive}+\mathrm{false}\,\mathrm{negative}}.$$

In the per-patient analysis, the judgment of whether the patient was correctly categorized by the clinician or the CNN model was based on the majority voting rule method (24, 25). The threshold value was defined as the lowest value to judge whether the clinician or the CNN model correctly classified a patient into one of the three categories, and it was set to at least 50% to ensure there is no more than one category proportion could exceed the threshold value. In the voting rule method, the clinician or the CNN model was considered to correctly categorize a patient when the proportion of the patient’s correctly classified images exceeded the threshold value, otherwise, the clinician or the CNN model was considered to miscategorize the patient. The categorization accuracy of a clinician or the CNN model was calculated as the proportion of patients considered to be correctly categorized by the clinician or the CNN model under different threshold values in the per-patient analysis.

Categorical variables were expressed as numbers in percentages. Continuous variables were expressed as the median of the interquartile range (IQR) if data were not normally distributed. All relevant data were analyzed using SPSS software, version 21.0.

## Results

### Characteristics of Subjects and Image Set

The clinical and demographic data of 217 CD patients, 279 UC patients, and 100 healthy controls were systematically documented and detailed in Table 1. Among them, the median (IQR) age of CD, UC, and healthy subjects were 28 (22–35), 45 (31–54), and 42 (34–51) years, respectively, while the proportion of male subjects in each group was 163 (75.12%), 179 (64.16%), and 56 (56.00%), respectively. Among the 217 CD patients, 115 (53.00%) were ileocolonic (115, 53.00%) and 96 (44.24%) were colonic, and 129 (59.45%) patients were non-stricturing and non-penetrating. Of the 279 UC patients, 142 (50.90%) had a pancolitis, 92 (32.97%) had a left-sided colitis, and 45 (16.13%) had a disease limited to the rectum.

**TABLE 1:** Demographic characteristics of CD patients, UC patients and healthy controls.

Variables	CD patients	UC patients	Healthy controls
	(*n* = 217)	(*n* = 279)	(*n* = 100)
Gender, *n* (%)			
Male	163 (75.12)	179 (64.16)	56 (56.00)
Female	54 (24.88)	100 (35.84)	44 (44.00)
Age, Median (IQR), y	28 (22–35)	45 (31–54)	42 (34–51)
Montreal classification, *n* (%)			
UC extent			
E1 Proctitis	NA	45 (16.13)	NA
E2 Left-sided colitis	NA	92 (32.97)	NA
E3 Extensive colitis	NA	142 (50.90)	NA
Age at diagnosis (A), *n* (%)			
A1 16 years or younger	18 (8.29)	NA	NA
A2 17–40 years	159 (73.27)	NA	NA
A3 Over 40 years	40 (18.43)	NA	NA
Location (L), *n* (%)			
L1 Terminal ileum	6 (2.76)	NA	NA
L2 Colon	96 (44.24)	NA	NA
L3 Ileocolon	115 (53.00)	NA	NA
L4 Upper GI	0	NA	NA
Behavior (B), *n* (%)			
B1 Non-stricturing, non-penetrating	129 (59.45)	NA	NA
B2 Stricturing	69 (31.80)	NA	NA
B3 Penetrating	19 (8.76)	NA	NA
P Perianal disease modifier	12 (5.5)	NA	NA

*CD, Crohn’s disease; UC, ulcerative colitis; IQR, interquartile range; GI, gastrointestinal; NA, not applicable.*

After image preprocessing, 42,267 images were excluded according to the exclusion criteria as mentioned previously. The remaining 15,330 eligible colonoscopy images, consisting of 5,111 active lesion images from 217 CD patients and 5,219 active lesion images from 279 UC patients and randomly extracted 5,000 normal images from a group of 100 healthy controls, were included to generate the image set in our study. Representative images that can be input to the CNN model are presented in Supplementary Figure 2.

### The Performance of the Convolutional Neural Network Model on the Three-Category Classification Task

In the three-category classification task of colonoscopy images from CD/UC patients and healthy controls, the CNN model achieved an overall accuracy of 92.04%. The detailed per-category performance of the established model has been presented in Table 2. The ResNeXt-101 showed a diagnostic accuracy of 92.39% for active CD lesion images, 93.35% for active UC lesion images, while it peaked at 98.35% for control images. The sensitivity, specificity, PPV, NPV and F1-score of the CNN model for classifying CD were 87.53, 94.78, 89.19, 93.91, and 0.88%, respectively; that for UC were 90.49, 98.14, 89.94, 95.11, and 0.90%, respectively; and for classifying control images were 98.14, 98.46, 96.93, 99.07, and 0.98%, respectively. The confusion matrix for the per-category sensitivity of the ResNeXt-101 in the test image set has been presented in Supplementary Figure 3A.

**TABLE 2:** Diagnostic performance of the CNN model and clinicians in classifying CD, UC or normal on endoscopic images in the test dataset.

	The CNN model	Clinician 1	Clinician 2	Clinician 3	Clinician 4	Clinician 5	Clinician 6
**CD**							
Accuracy	92.39 (90.88–93.67)	91.70 (90.14–93.04)	81.28 (79.16–83.23)	87.24 (85.39–88.89)	78.53 (76.31–80.59)	73.53 (71.17–75.76)	86.90 (85.03–88.57)
Sensitivity	87.53 (84.16–90.28)	86.28 (82.80–89.16)	76.92 (72.84–80.56)	65.49 (61.03–69.70)	36.38 (32.10–40.88)	26.82 (22.96–31.06)	80.04 (76.13–83.46)
Specificity	94.78 (93.14–96.05)	94.37 (92.69–95.69)	83.42 (80.90–85.67)	97.95 (96.79–98.71)	99.28 (98.46–99.68)	96.52 (95.12–97.54)	90.28 (88.21–92.03)
PPV	89.19 (85.95–91.77)	88.30 (84.96–90.99)	69.55 (65.41–73.40)	94.03 (90.78–96.22)	96.15 (91.91–98.30)	79.14 (71.94–84.94)	80.21 (76.30–83.62)
NPV	93.91 (92.18–95.28)	93.32 (91.53–94.76)	88.01 (85.70–90.00)	85.22 (82.98–87.22)	90.15 (88.17–91.83)	72.82 (70.29–75.21)	90.18 (88.10–91.94)
F1–score	0.88 (0.85–0.91)	0.87 0.84–0.90)	0.73 (0.69–0.77)	0.77 (0.73–0.81)	0.53 (0.48–0.58)	0.40 (0.35–0.45)	0.80 (0.76–0.84)
**UC**							
Accuracy	93.35 (91.92–94.55)	92.39 (90.88–93.67)	79.84 (77.67–81.85)	90.26 (88.59–91.71)	83.20 (81.16–85.06)	59.60 (57.02–62.12)	86.76 (84.89–88.44)
Sensitivity	90.49 (87.47–92.86)	92.91 (90.19–94.94)	67.81 (63.46–71.88)	92.51 (89.73–94.60)	84.62 (81.06–87.63)	96.36 (94.20–97.76)	80.57 (76.74–83.91)
Specificity	94.81 (93.17–96.09)	92.12 (90.19–93.71)	86.00 (83.61–8810)	89.11 (86.93–90.97)	82.47 (79.89–84.79)	40.77 (37.66–43.96)	89.94 (87.82–91.73)
PPV	89.94 (86.87–92.37)	85.79 (82.48–88.58)	71.28 (66.92–75.29)	81.32 (77.79–84.41)	71.21 (67.33–74.81)	45.46 (42.42–48.54)	80.40 (76.57–83.75)
NPV	95.11 (93.50–96.35)	96.21 (94.71–97.31)	83.91 (81.43–86.12)	95.87 (94.30–97.04)	91.27 (89.15–93.02)	95.62 (93.40–97.31)	90.03 (87.92–91.81)
F1–score	0.90 (0.87–0.93)	0.89 (0.86–0.92)	0.70 (0.65–0.74)	0.87 (0.83–0.89)	0.77 (0.74–0.81)	0.62 (0.58–0.65)	0.80 (0.77–0.84)
**Normal**							
Accuracy	98.35 (97.52–98.92)	97.26 (96.25–98.01)	95.54 (94.32–96.52)	94.65 (93.34–95.72)	85.60 (83.67–87.34)	83.47 (81.44–85.32)	98.77 (98.02–99.25)
Sensitivity	98.14 (96.37–99.09)	92.75 (89.97–94.83)	90.48 (87.42–92.88)	100 (99.02–100)	99.59 (98.35–99.93)	50.72 (46.17–55.26)	98.14 (96.37–99.09)
Specificity	98.46 (97.41–99.10)	99.49 (98.74–99.81)	98.05 (96.91–98.79)	92.00 (90.07–93.59)	78.67 (75.94–81.17)	99.69 (99.02–99.92)	99.08 (98.19–99.55)
PPV	96.93 (94.87–98.21)	98.90 (97.30–99.60)	95.83 (93.45–97.40)	86.10 (82.89–88.80)	69.81 (66.21–73.19)	98.79 (96.21–99.69)	98.14 (96.37–99.09)
NPV	99.07 (98.18–99.55)	96.52 (95.14–97.53)	95.41 (93.88–96.58)	100 (99.47–100)	99.74 (98.96–99.95)	80.33 (77.95–82.51)	99.08 (98.19–99.55)
F1–score	0.98 (0.96–0.99)	0.96 (0.93–0.97)	0.93 (0.90–0.95)	0.93 (0.90–0.94)	0.82 (0.79–0.84)	0.67 (0.62–0.71)	0.98 (0.96–0.99)
**Overall accuracy**	92.04 (90.50–93.35)	90.67 (89.03–92.09)	78.33 (76.11–80.40)	86.08 (84.17–87.79)	73.66 (71.30–75.89)	58.30 (55.72–60.84)	86.21 (84.31–87.92)

*All results are given as a percentage (95% CI).*

*CNN, convolutional neural network; CD, Crohn’s disease; UC, ulcerative colitis; CI, confidence interval; PPV, positive predictive value; NPV, negative predictive value.*

### Comparison of Performances Between the Clinicians and the Artificial Intelligence-Guided Convolutional Neural Network Model

Among the 1,458 test images, the overall accuracy of each of the six clinicians was 90.67, 78.33, 86.08, 73.66, 58.30, and 86.21%, respectively, in classifying the colonoscopy images from CD, UC, and healthy subjects, which were lower than overall accuracy of 92.04% of the CNN model (Table 2). For the classification of CD lesion images, the clinician with the best performance showed an accuracy of 91.70%, a sensitivity of 86.28%, and a specificity of 94.37%, which were all inferior to those of the CNN model. The clinician with the best performance also achieved a slightly lower classification accuracy when compared with that of the CNN model (92.39 vs. 93.35% for UC lesion images; 97.26 vs. 98.35% for control images). Besides, the F1-score of clinicians in each category was inferior to that of the CNN model. Generally, the CNN model showed improved overall performance and reproducibility in the classification task. The confusion matrices of the clinicians have been shown in Supplementary Figure 3.

According to the results of per-patient analysis, clinicians of different seniority achieved an accuracy of 93.94, 78.79, 83.33, 59.09, 56.06, and 90.91%, respectively, in conventional reading, while the CNN model achieved an accuracy of 90.91% under the 50% threshold value (Table 3). Based on these results, it can be suggested that the CNN model might be better than most clinicians but not the best when classifying individual patients. In other words, the performance of the CNN model was somewhat inferior to the experienced experts to a certain extent.

**TABLE 3:** Diagnostic accuracy of the CNN model and clinicians in per-patient analysis.

Threshold value	The CNN model	Clinician 1	Clinician 2	Clinician 3	Clinician 4	Clinician 5	Clinician 6
50%	90.91 (80.62–96.25)	93.94 (84.44–98.04)	78.79 (66.66–87.52)	83.33 (71.71–90.99)	59.09 (46.30–70.82)	56.06 (43.35–68.07)	90.91 (80.62–96.25)
60%	87.88 (76.96–94.25)	93.94 (84.44–98.04)	69.70 (57.00–80.09)	81.82 (70.01–89.96)	59.09 (46.30–70.82)	50.00 (37.56–62.44)	86.36 (75.18–93.19)
70%	86.36 (75.18–93.19)	89.39 (78.77–95.27)	63.64 (50.82–74.86)	75.76 (63.38–85.11)	53.03 (40.43–65.27)	46.97 (34.73–59.57)	77.27 (65.01–86.32)
80%	77.27 (65.01–86.32)	81.82 (70.01-89.86)	53.03 (40.43—-65.27)	66.67 (53.89–77.50)	46.97 (34.73–59.57)	40.91 (29.18–53.70)	63.64 (50.82–74.86)
90%	60.61 (47.80–72.18)	68.18 (55.43–78.80)	36.36 (25.14–49.18)	54.55 (41.89–66.68)	40.91 (29.18–53.70)	36.36 (25.14–49.18)	50.00 (37.56–62.44)

*All results are given as a percentage (95% CI).*

*CNN, convolutional neural network; CI, confidence interval.*

### Analysis of Misclassified Endoscopic Images

**FIGURE 3:**
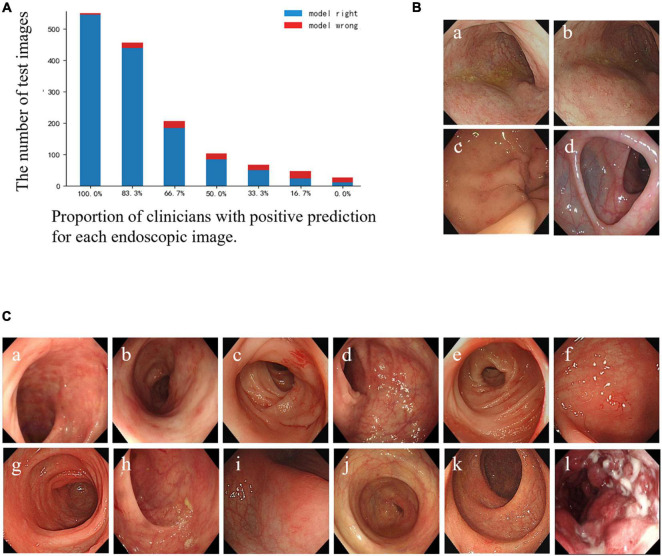
Comparison of prediction results between the CNN model and clinicians for each test image. **(A)** Bar diagram of the comparison of results between clinicians and the CNN model. The horizontal axis represents a corresponding proportion of the number of clinicians who classified the images correctly, and the corresponding quantities of the images are shown according to the height of the column and the number on the longitudinal axis. The blue column: the CNN model’s prediction is correct; the red column: the CNN model’s prediction is wrong. **(B)** Illustration of misclassified images by the CNN model, but not by the participating clinicians: (a–c) UC images misclassified as CD. (d) Normal images misclassified as CD. **(C)** Illustration of images that were misclassified by all clinicians, but not by the CNN model: (a–c) CD images misclassified as UC by all clinicians; (d–k) CD images misclassified as UC by most clinicians and misclassified as normal images by the rest; (l) UC misclassified as CD by all clinicians. CD, Crohn’s disease; UC, ulcerative colitis; CNN, convolutional neural network.

The specific misclassified condition of clinicians and the CNN model is shown in Figure 3A. According to the results, there were 3 UC images and 1 normal image misclassified as CD images by the CNN model, but not by the clinicians (Figure 3B). Furthermore, 12 images were mispredicted by all clinicians, while those were correctly predicted by the CNN model. Among them, 11 CD lesion images were misclassified as UC by most clinicians (Figure 3C). In general, clinicians were more inclined to classify active CD lesion images into UC, while the CNN model tended to misdiagnose UC lesion images as CD lesions. We speculated that the CNN model might correct the clinicians’ bias toward misclassification of CD as UC to some extent, but there was an overcorrection situation.

## Discussion

In the present study, we developed and validated a superior AI-based model using the ResNeXt-101 network to precisely classify between CD, UC, and normal colonoscopy images. Despite the significant importance of AI application in clinical image analysis, the deep learning technology has not been routinely applied in the classification and distinction of colonoscopy images of CD, UC, and healthy individuals. According to the results of the per-image classification analysis, the CNN model showed a superior overall accuracy compared with that of the six clinicians. Although it outperformed most clinicians, however, the accuracy of expert clinician (93.94%) was comparatively higher than that of the CNN model (90.91%) while making the diagnosis based on all enrolled endoscopic images of certain patients. Thus, it indicates that the CNN model has excellent classification performance in the task, and could be an instructive and powerful tool to assist the inadequately experienced clinicians.

Although endoscopy plays a pivotal role in the diagnosis of IBD, diagnostic precision highly depends on the technical skills and extensive experiences of the operators. The most typical endoscopic features of UC are continuous and confluent colonic involvement with clear demarcation of inflammation and mucosal friability, while CD is characterized by discontinuous lesions, longitudinal ulcers, cobblestone appearance, the presence of stricture, fistulas, or perianal involvement (26). It is not an easy task to identify different forms of IBD based on the endoscopic images alone, even for the experts, due to the exclusivity and comprehensiveness of the diagnosis of IBD. Besides, there has been a shortage of expert gastroenterologists in the field of IBD. In addition, professional IBD training for junior clinicians has not yet been popularized. Thus, intelligent adjunctive tools with promising applications in clinical image analysis could potentially facilitate effective endoscopic diagnosis.

AI has recently been applied to the research of IBD diagnosis. For example, Hubenthal et al. used a penalized support vector machine for analyzing microarray-based miRNA expression profiles from peripheral blood samples to achieve a differential diagnosis of CD and UC with a remarkably small classification error rate of 3.1%. However, the generalizability of the model to other technologies was limited as the model was trained based on the same type of data originated from the Geniom Array (27). Mossotto et al. developed a supervised model based on the support vector machine utilizing combined endoscopic and histological disease location data to classify pediatric IBD with a diagnostic accuracy of 82.7% (28). Furthermore, Tong et al. built a classifier by random forest to differentiate between CD and UC based on the endoscopic results in the form of free text rather than images, which yielded sensitivity and specificity of 89 and 84%, respectively (29). Although previous studies had a certain reference for further investigation, each had different footholds. Importantly, our study focused on applying the CNN to the intelligent processing of a large set of colonoscopy images to establish a practicable model. Furthermore, clinicians of different levels of seniority performed the classification task and their performances were evaluated, which increased the generalizability of the study. In addition to the state-of-the-art deep learning architecture of the model, our CNN model included several other user-friendly interfaces in terms of its simplicity, feasibility, and cost-effectivity, since the model required only the input of original clinical data, such as endoscopic images.

Thus, our study demonstrates the superb practical applicability of deep learning techniques in managing the mass image data, with a robust accuracy at a level equivalent to or better than that of professional clinicians. This study set the path for the further exploration of integrating endoscopic images with multimodal clinical data to construct a combined model with significantly higher efficiency and accuracy. This was a pilot exploration toward clinical translation of this method. It is worth evaluating the classification capability of the CNN model with images at the initial stage, although it would not be sufficient to train the model in the sense that endoscopic still images could not reflect the actual situations that the model would encounter in reality. A dataset containing colonoscopy video recordings or a similar clinical setting must be implemented to confirm the applicability of the CNN model in precisely analyzing gastrointestinal images. Following that, we would go into the details of achieving real-time use of AI during endoscopic diagnoses, such as the interpretation of the endoscopic images of IBD, integration of additional clinical information as required, discrimination of the lesion types, guiding the suspicious lesion’s biopsy, the differential diagnosis of IBD mimics, etc.

An empirical analysis was performed for the misclassification of the task by clinicians, and the major decision-influencing factors were as follows: (1) images of mild lesions from CD and UC patients shared similar characteristics; (2) some images of patients with severe UC or CD lesions were prone to be misclassified; (3) the area of the lesion was so limited in the field of view that it could easily be overlooked, etc. Even though the CNN model could not achieve absolute accuracy, it still exhibited the satisfactory performance in the distinction between CD, UC, and control images at the colonoscopy image level. As observed in our investigation, the CNN model was more sensitive than clinicians in identifying CD lesions in endoscopic images, but at the same time, it yielded some false positives. The tendency of the CNN model to overcorrect the clinicians’ judgment suggests the necessity for further training of our model with more diverse and larger sets of clinical images to improve its clinical applicability.

Despite multiple positive aspects of our CNN model in IBD image analysis and diagnosis, our study suffers from certain limitations that should be discussed to better understand the potential avenues to improve the model further. Firstly, the CNN model was developed and tested in retrospective datasets. Secondly, our model may had been overfitted, concerning our limited image set size and the absence of images from IBD mimics. So, larger prospective cohorts of subjects including IBD and IBD mimics need to be enrolled in future studies to precisely optimize the model before its real-life clinical application. Thirdly, all enrolled endoscopic images were Olympus images, so it is necessary to test the CNN model on images captured by endoscopes from other manufacturers (e.g., Pentax, Fujifilm, etc.). Additionally, stratification of the image by the CD or UC endoscopic scoring system and more clinical information were not included in this CNN model. Finally, the images with normal endoscopic features in CD or UC patients were not included in the per-patient analysis test set, which did not fully simulate the real clinical situation in CD and UC colonoscopy diagnosis. Further studies need to be conducted to assess the performance of per-patient colonoscopy images analysis based on the whole colonoscopy images of each patient. With statistically larger and better-designed prospective trials, this novel technology for gastrointestinal endoscopy-based diagnosis of IBD may be implemented in clinical practice soon.

## Conclusion

In conclusion, the CNN model performed superior to most clinicians in the blind review of active CD/UC lesion images from the respective patients and normal images from healthy subjects in per-image and per-patient analyses, suggesting that the CNN model can assist most clinicians in the three-category classification task. Therefore, we will further improve the CNN model by increasing the diversity of the test datasets and preferably incorporating clinical data to make this model better suitable in clinical settings.

## Data Availability Statement

The raw data supporting the conclusions of this article will be made available by the authors, without undue reservation.

## Ethics Statement

The studies involving human participants were reviewed and approved by the Ethical Committee of Tongji Hospital, Tongji Medical College, Huazhong University of Science and Tongji Medical College, Huazhong University of Science and Technology. Written informed consent from the participants or their legal guardian/next of kin was not required to participate in this study in accordance with the national legislation and the institutional requirements.

## Author Contributions

FX designed the study. LW and FX drafted the manuscript. LC and XW collected and reviewed the data. LW, LC, JH, SX, JX, FX, and XW analyzed the clinical data. LW, KL, TL, and YY performed the figure and table preparation. FX, DT, and US revised the manuscript. All authors read and approved the final manuscript.

## Conflict of Interest

XW, KL, TL, and YY were employed by Wuhan United Imaging Healthcare Surgical Technology Co., Ltd. The remaining authors declare that the research was conducted in the absence of any commercial or financial relationships that could be construed as a potential conflict of interest.

## Publisher’s Note

All claims expressed in this article are solely those of the authors and do not necessarily represent those of their affiliated organizations, or those of the publisher, the editors and the reviewers. Any product that may be evaluated in this article, or claim that may be made by its manufacturer, is not guaranteed or endorsed by the publisher.

## Abbreviations

AI: artificial intelligence
CD: Crohn’s disease
CNN: convolutional neural network
IBD: inflammatory bowel disease
UC: ulcerative colitis.

## References

[1] Li X Song P Li J Tao Y Li G Li X The disease burden and clinical characteristics of inflammatory bowel disease in the Chinese population: a systematic review and meta-analysis. *Int J Environ Res Public Health.* (2017) 14:238. 10.3390/ijerph14030238 28264519 PMC5369074

[2] Ng SC Shi HY Hamidi N Underwood FE Tang W Benchimol EI Worldwide incidence and prevalence of inflammatory bowel disease in the 21st century: a systematic review of population-based studies. *Lancet.* (2017) 390:2769–78. 10.1016/S0140-6736(17)32448-0 29050646

[3] Banerjee R Pal P Mak JWY Ng SC. Challenges in the diagnosis and management of inflammatory bowel disease in resource-limited settings in Asia. *Lancet Gastroenterol Hepatol.* (2020) 5:1076–88. 10.1016/S2468-1253(20)30299-5 33181087

[4] Tontini GE Vecchi M Pastorelli L Neurath MF Neumann H. Differential diagnosis in inflammatory bowel disease colitis: state of the art and future perspectives. *World J Gastroenterol.* (2015) 21:21–46. 10.3748/wjg.v21.i1.21 25574078 PMC4284336

[5] Magro F Gionchetti P Eliakim R Ardizzone S Armuzzi A Barreiro-de Acosta M Third European evidence-based consensus on diagnosis and management of ulcerative colitis. Part 1: definitions, diagnosis, extra-intestinal manifestations, pregnancy, cancer surveillance, surgery, and ileo-anal pouch disorders. *J Crohns Colitis.* (2017) 11:649–70. 10.1093/ecco-jcc/jjx008 28158501

[6] Gomollon F Dignass A Annese V Tilg H Van Assche G Lindsay JO 3rd European evidence-based consensus on the diagnosis and management of crohn’s disease 2016: part 1: diagnosis and medical management. *J Crohns Colitis.* (2017) 11:3–25. 10.1093/ecco-jcc/jjw168 27660341

[7] Nunez FP Krugliak Cleveland N Quera R Rubin DT. Evolving role of endoscopy in inflammatory bowel disease: going beyond diagnosis. *World J Gastroenterol.* (2021) 27:2521–30. 10.3748/wjg.v27.i20.2521 34092973 PMC8160621

[8] Buchner AM. Confocal laser endomicroscopy in the evaluation of inflammatory bowel disease. *Inflamm Bowel Dis.* (2019) 25:1302–12. 10.1093/ibd/izz021 30877772

[9] Maeda Y Kudo SE Mori Y Misawa M Ogata N Sasanuma S Fully automated diagnostic system with artificial intelligence using endocytoscopy to identify the presence of histologic inflammation associated with ulcerative colitis (with video). *Gastrointest Endosc.* (2019) 89:408–15. 10.1016/j.gie.2018.09.024 30268542

[10] Hewett DG Kahi CJ Rex DK. Efficacy and effectiveness of colonoscopy: how do we bridge the gap? *Gastrointest Endosc Clin N Am.* (2010) 20:673–84. 10.1016/j.giec.2010.07.011 20889071

[11] Fierson WM American Academy of Pediatrics Section on Ophthalmology [AAP], American Academy of Ophthalmology [AAO], American Association for Pediatric Ophthalmology and Strabismus [AAPOS], American Association of Certified Orthoptists [AACO]. Screening examination of premature infants for retinopathy of prematurity. *Pediatrics.* (2018) 142:e20183061. 10.1542/peds.2018-3810 30478242

[12] Murdoch TB Detsky AS. The inevitable application of big data to health care. *JAMA.* (2013) 309:1351–2. 10.1001/jama.2013.393 23549579

[13] Esteva A Kuprel B Novoa RA Ko J Swetter SM Blau HM Dermatologist-level classification of skin cancer with deep neural networks. *Nature.* (2017) 542:115–8. 10.1038/nature21056 28117445 PMC8382232

[14] Tong Y Lu W Deng QQ Chen C Shen Y. Automated identification of retinopathy of prematurity by image-based deep learning. *Eye Vis (Lond).* (2020) 7:40. 10.1186/s40662-020-00206-2 32766357 PMC7395360

[15] Remedios LW Lingam S Remedios SW Gao R Clark SW Davis LT Technical note: comparison of convolutional neural networks for detecting large vessel occlusion on computed tomography angiography. *Med Phys.* (2021) 48:6060–8. 10.1002/mp.15122 34287944 PMC8568625

[16] Mccarthy J Minsky ML Rochester N Shannon CE. A proposal for the Dartmouth summer research project on artificial intelligence. *AI Mag.* (1955) 27:12.

[17] LeCun Y Bengio Y Hinton G. Deep learning. *Nature.* (2015) 521:436–44. 10.1038/nature14539 26017442

[18] Kumar PR Manash EBK. Deep learning: a branch of machine learning. *J Phys Conf Ser.* (2019) 1228:012045.

[19] Rintaro H James R Tyler D Andrew N Elise T Daniel M Artificial intelligence using convolutional neural networks for real-time detection of early esophageal neoplasia in Barrett’s esophagus (with video). *Gastrointest Endosc.* (2020) 91:1264–71.e1. 10.1016/j.gie.2019.12.049 31930967

[20] Namikawa K Hirasawa T Nakano K Ikenoyama Y Ishioka M Shiroma S Artificial intelligence-based diagnostic system classifying gastric cancers and ulcers: comparison between the original and newly developed systems. *Endoscopy.* (2020) 52:1077–83. 10.1055/a-1194-8771 32503056

[21] Urban G Tripathi P Alkayali T Mittal M Jalali F Karnes W Deep learning localizes and identifies polyps in real time with 96% accuracy in screening colonoscopy. *Gastroenterology.* (2018) 155:1069–78.e8. 10.1053/j.gastro.2018.06.037 29928897 PMC6174102

[22] Goodfellow I Bengio Y Courville A. *Deep Learning.* Cambridge: MIT Press (2016).

[23] Xie J Zhu SC Wu YN. Learning energy-based spatial-temporal generative convnets for dynamic patterns. *IEEE Trans Pattern Anal Mach Intell.* (2021) 43:516–31. 10.1109/TPAMI.2019.2934852 31425020

[24] Tao D Cheng J Yu Z Yue K Wang L. Domain-weighted majority voting for crowdsourcing. *IEEE Trans Neural Netw Learn Syst.* (2019) 30:163–74. 10.1109/TNNLS.2018.2836969 29994339

[25] Li X Huang H Zhang J Jiang F Guo Y Shi Y A qualitative transcriptional signature for predicting the biochemical recurrence risk of prostate cancer patients after radical prostatectomy. *Prostate.* (2020) 80:376–87. 10.1002/pros.23952 31961962 PMC7065139

[26] Maaser C Sturm A Vavricka SR Kucharzik T Fiorino G Annese V ECCO-ESGAR guideline for diagnostic assessment in IBD part 1: initial diagnosis, monitoring of known IBD, detection of complications. *J Crohns Colitis.* (2019) 13:144–64. 10.1093/ecco-jcc/jjy113 30137275

[27] Hubenthal M Hemmrich-Stanisak G Degenhardt F Szymczak S Du Z Elsharawy A Sparse modeling reveals miRNA signatures for diagnostics of inflammatory bowel disease. *PLoS One.* (2015) 10:e0140155. 10.1371/journal.pone.0140155 26466382 PMC4605644

[28] Mossotto E Ashton JJ Coelho T Beattie RM Macarthur BD Ennis SJSR. Classification of paediatric inflammatory bowel disease using machine learning. *Sci Rep.* (2017) 7:2427. 28546534 10.1038/s41598-017-02606-2 PMC5445076

[29] Tong Y Lu K Yang Y Li J Lin Y Wu D Can natural language processing help differentiate inflammatory intestinal diseases in China? Models applying random forest and convolutional neural network approaches. *BMC Med Inform Decis Mak.* (2020) 20:248. 10.1186/s12911-020-01277-w 32993636 PMC7526202

