# Supplementary material for: Development of a Convolutional Neural Network-Based Colonoscopy Image Assessment Model for Differentiating Crohn’s Disease and Ulcerative Colitis
Source: Front Med (Lausanne). 2022 Apr 8;9:789862. doi: 10.3389/fmed.2022.789862 (PMC9024394; doi:10.3389/fmed.2022.789862)
Supplement: Supplementary file 1 [file Data_Sheet_1.PDF]

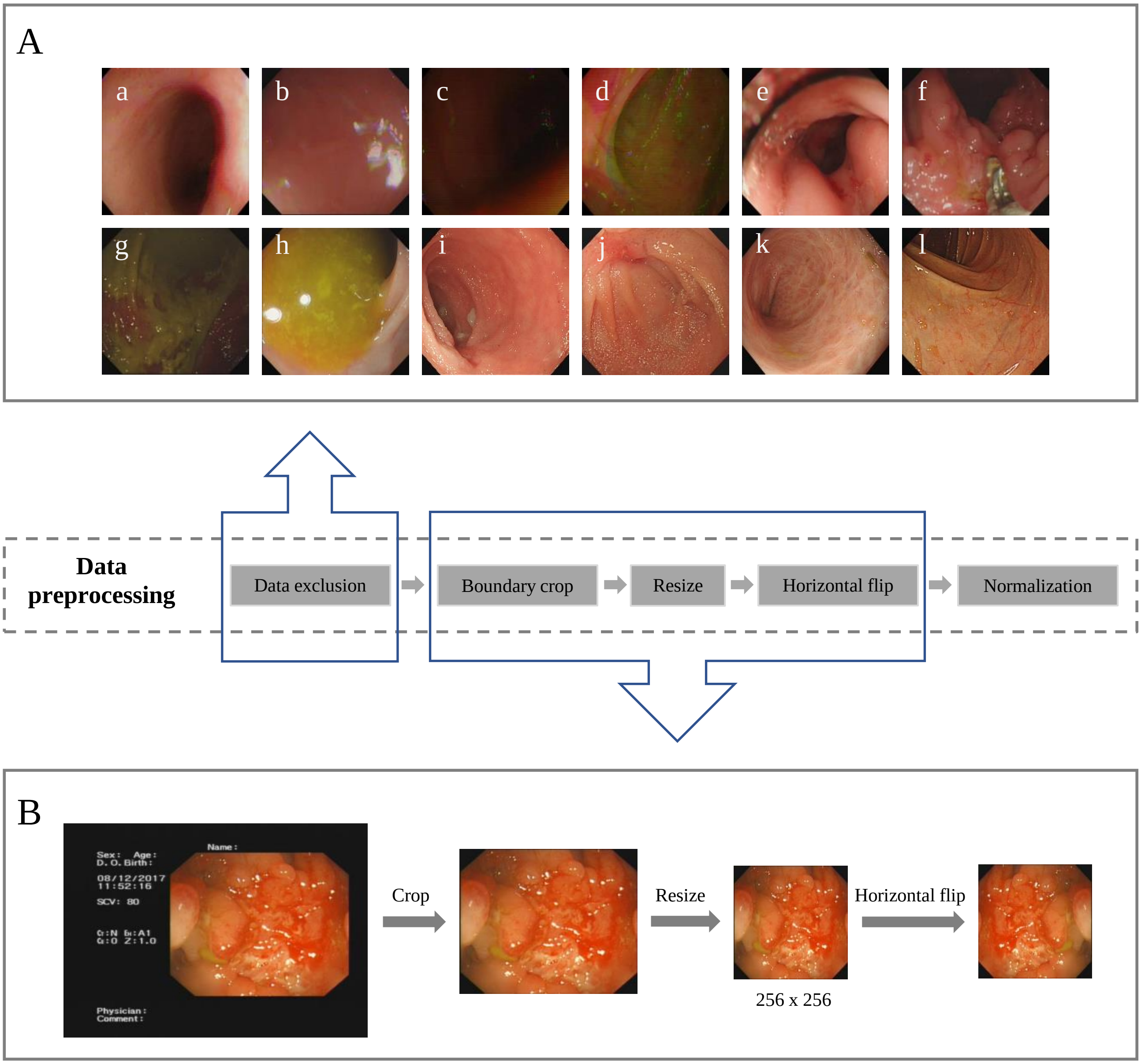

Supplementary Figure 1. Illustration of multiple data preprocessing operations: The preprocessed work performed toward raw data including data exclusion, boundary cropping, resizing, etc. (A) Examples of excluded non-informative images: (a-f) Representative illustration of images with poor quality: (a) motion-blurring, (b) out-of-focus, (c) improper lighting, (d) glare, (e) droplet or spout, (f) disturbed by endoscope or accessories, (g-h) poor bowel preparation, (i-j) small intestine fragments, (k-l) images of inactive IBD patients. (B) A simple demonstration of the processes of an original image being cropping, resizing, and flipping horizontally.

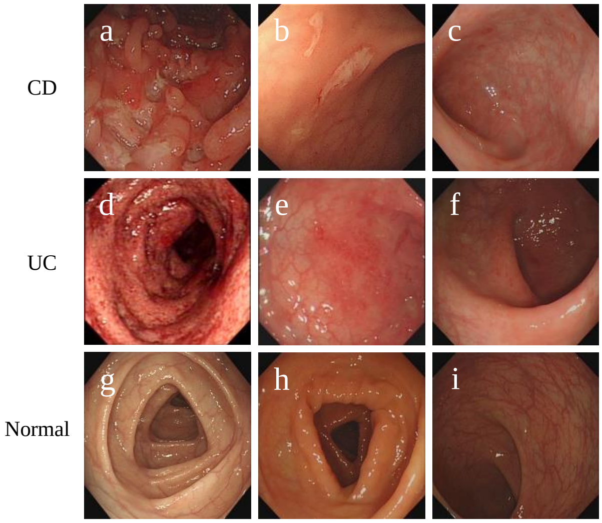

Supplementary Figure 2. Representative endoscopic images of each classification. (a-c) CD images of varying severity; (d-f) UC images of varying severity, and (g-i) normal images of different gastrointestinal fragments. CD, Crohn's disease; UC, ulcerative colitis.

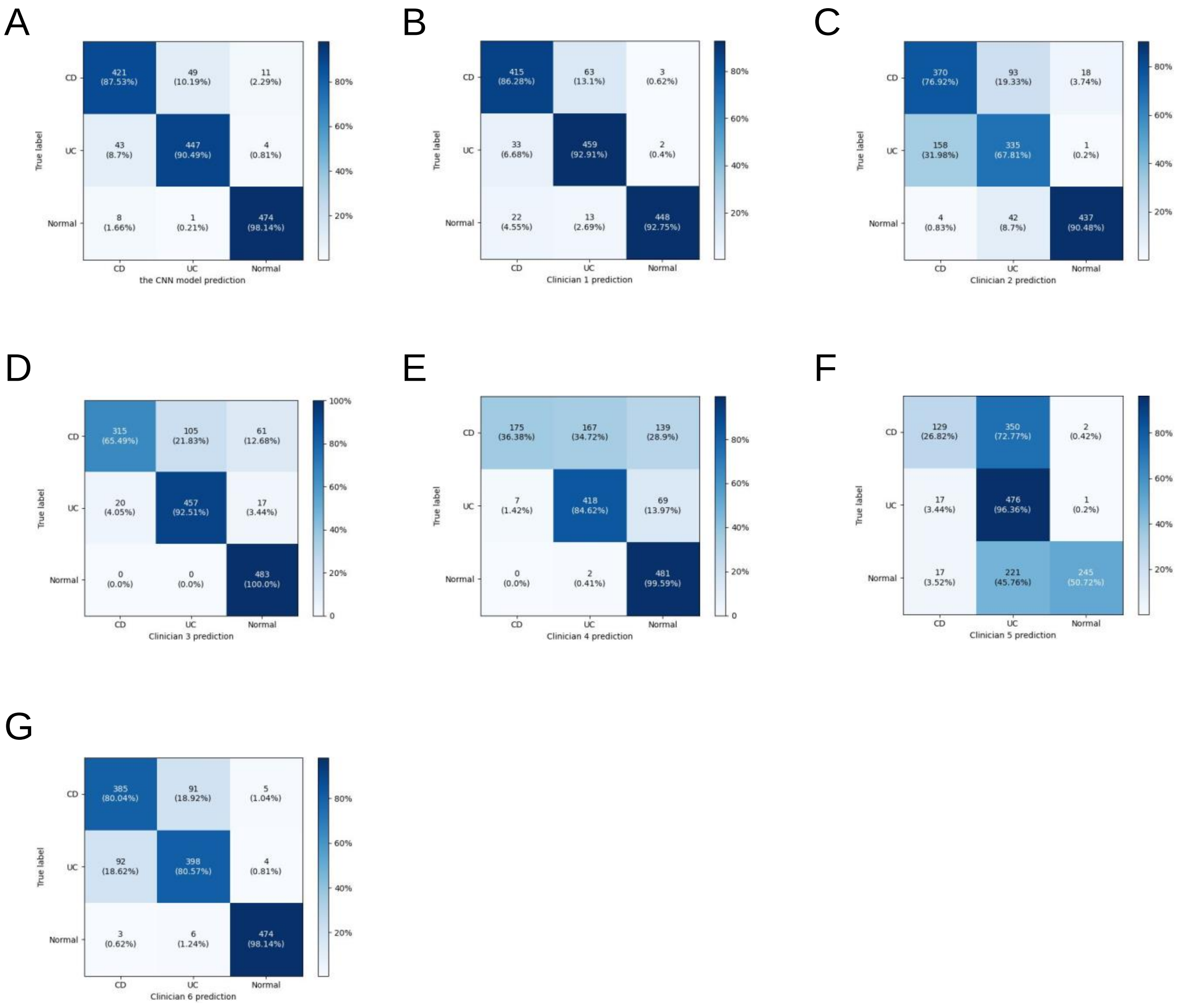

Supplementary Figure 3. Seven confusion matrices for the CNN model' and clinicians' predictions in the three-classification task. (A) Confusion matrix of the CNN model; (B) Confusion matrix of clinician 1; (C) Confusion matrix of clinician 2; (D) Confusion matrix of clinician 3; (E) Confusion matrix of clinician 4; (F) Confusion matrix of clinician 5; (G) Confusion matrix of clinician 6.
